# Benthic communities in three Mediterranean touristic ports: MAPMED project

**DOI:** 10.3897/BDJ.9.e66420

**Published:** 2021-04-26

**Authors:** Eva Chatzinikolaou, Panagiotis Damianidis, Christina Pavloudi, Aikaterini Vasileiadou, Sarah Faulwetter, Kleoniki Keklikoglou, Wanda Plaitis, Dimitra Mavraki, Stamatina Nikolopoulou, Christos Arvanitidis

**Affiliations:** 1 Hellenic Centre for Marine Research (HCMR), Institute of Marine Biology, Biotechnology and Aquaculture (IMBBC), Heraklion, Crete, Greece Hellenic Centre for Marine Research (HCMR), Institute of Marine Biology, Biotechnology and Aquaculture (IMBBC) Heraklion, Crete Greece; 2 Aristotle University of Thessaloniki, Thessaloniki, Greece Aristotle University of Thessaloniki Thessaloniki Greece; 3 Hellenic Centre for Marine Research (HCMR), Institute of Oceanography, Athens, Greece Hellenic Centre for Marine Research (HCMR), Institute of Oceanography Athens Greece; 4 Biology Department, University of Crete, Heraklion, Crete, Greece Biology Department, University of Crete Heraklion, Crete Greece; 5 LifeWatch ERIC, Seville, Spain LifeWatch ERIC Seville Spain

**Keywords:** macrobenthos, ports, harbours, Mediterranean, Greece, Italy, Tunisia

## Abstract

**Background:**

Mediterranean ports are sources of significant economic activity and at the same time they act as recipients of considerable anthropogenic disturbance and pollution. Polluted and low-in-oxygen sediments can negatively impact benthic biodiversity and favour recruitment of opportunistic or invasive species. Macrobenthic communities are an important component of the port biota and can be used as environmental quality indicators. However, a baseline database for benthic biodiversity in Mediterranean ports has not yet been widely established.

**New information:**

Macrobenthic assemblages were recorded in three Mediterranean touristic ports under the framework of the ENPI CBC MED project MAPMED (MAnagement of Port Areas in the MEDiterranean Sea Basin). Samples were collected from Cagliari (Sardinia, Italy), Heraklion (Crete, Greece) and El Kantaoui (Tunisia) ports during February, May and September 2012. The sampling stations were selected according to the different sectors within each port (i.e. leisure, fishing, passenger/cargo vessels and shipyard). A total of 277 taxa belonging to 12 phyla were found, of which the 96 taxa were present in all three ports. El Kantaoui port hosted the highest number of macrobenthic taxa. Mollusca were the most abundant group (34%) in all ports. The highest percentage of opportunistic taxa per station was found before the touristic period in the shipyard of Heraklion port (89.3%).

## Introduction

Mediterranean ports are sources of significant economic activity and they strongly support local, regional and national economic development. The Mediterranean Sea hosts about 480 ports and terminals and is one of the busiest maritime areas of the world (REMPEC 2008). Shipping of goods between the main EU ports and ports located in the Mediterranean reached 598 million tonnes in 2015 (Eurostat 2015), while crude oil transported through the Mediterranean Sea was 421 million tonnes in 2006 (REMPEC 2008). Ports act as recipients of considerable anthropogenic disturbance and pollution due to the activities they are hosting, such as emission of air pollutants, noise, sediment dredging and transport, industrial installations, wastewater discharges, oil spill accidents, storage and spillage of hazardous materials and introduction of invasive species (Darbra et al. 2005). Polluted marine sediments, commonly low oxygen levels and low hydrodynamism can have a negative impact on benthic biota and marine biodiversity, which may favour recruitment by opportunistic or more resistant taxa, including invasive species. Macrobenthic communities are an important component of the port biota and have been commonly used as environmental quality indicators in biomonitoring studies (Gray and Elliot 2010). Therefore, the establishment of a baseline database for benthic biodiversity in Mediterranean ports can offer valuable background information for port management activities, including the identification of biological risks, such as pollution events or invasion by alien species (Mandal and Harkantra 2013).

## General description

### Purpose

This dataset includes macrobenthic taxa identified in three touristic ports in the Mediterranean, located at Cagliari (Sardinia, Italy), Heraklion (Crete, Greece) and El Kantaoui (Tunisia) (Chatzinikolaou and Arvanitidis 2017). Sampling was undertaken seasonally during the ENPI CBC MED project MAPMED in 2012 in different sectors within each port, which were defined according to their distinct usage activities (i.e. leisure, fishing, passenger/cargo vessels and shipyard). Samples were collected during winter (February), before the touristic period (May) and after the touristic period (September) in order to identify the impact of seasonal touristic activities on benthic communities. A detailed comparison of macrobenthic biodiversity amongst different locations - sectors - seasons was performed in order to offer information about the environmental quality at these understudied artificial port environments. A total of 277 taxa belonging to 12 phyla were found, of which 96 taxa were common in all ports. Differences in benthic biodiversity were found between ports and between sectors in each port, while seasonal differences were not apparent (Chatzinikolaou et al. 2018). The El Kantaoui port hosted the highest number of macrobenthic taxa, while the shipyard sector in Heraklion port had the lowest number of taxa. The highest abundance of opportunistic taxa was found in Heraklion port at the passenger ships and shipyard stations.

## Project description

### Title

MAPMED: MAnagement of Port areas in the MEDiterranean sea basin

### Personnel

Dr Eva Chatzinikolaou (project management, design and implementation of sampling, taxonomic identification), Dr Panagiotis Damianidis (taxonomic identification), Dr Christina Pavloudi (taxonomic identification), Dr Katerina Vasileiadou (taxonomic identification), Dr Sarah Faulwetter (taxonomic identification), Kleoniki Keklikoglou (taxonomic identification), Wanda Plaiti (taxonomic identification), Dimitra Mavraki (data management), Stamatina Nikolopoulou (data management) and Dr Christos Arvanitidis (principal investigator).

### Study area description

Mediterranean touristic ports: Cagliari (Sardinia, Italy), Heraklion (Crete, Greece) and El Kantaoui (Tunisia).

### Design description

During this study, differences in macrobenthic assemblages were examined across three levels: a) geographical differences (three countries), b) use-sectoral differences (3-5 stations within ports) and c) temporal differences (three seasons in relation to the touristic period).

### Funding

This project has been financed by the European Union under the ENPI CBC Mediterranean Sea Basin Programme in the framework of the project “MAnagement of Port areas in the MEDiterranean Sea Basin” (MAPMED) (Grand Contract 6/2019-25/ 7/2011). This publication has been supported by the LifeWatchGreece infrastructure (MIS 384676), funded by the Greek Government under the General Secretariat of Research and Technology (GSRT), ESFRI Projects, National Strategic Reference Framework (NSRF) and from LifeWatch ERIC.

## Sampling methods

### Sampling description

Five replicates of sediment samples were collected from each station using a box corer (13.5 cm × 13.5 cm × 16 cm) manually operated from a small boat. A Garmin 60 CS portable GPS and a depth meter were available on board to record the exact position and depth of each station respectively. The sediment samples were sieved through a 0.5 mm sieve and then fixed and preserved in 5% formaldehyde buffered with seawater and stained with Rose Bengal. The benthic organisms were sorted out of the sediment under a stereoscope, counted and identified down to the lowest possible taxonomic level.

### Quality control

All scientific names were standardised against the World Register of Marine Species using the Taxon Match tool (http://www.marinespecies.org/aphia.php?p=match) on 10-03-2021.

## Geographic coverage

### Description

**Figure 1.**
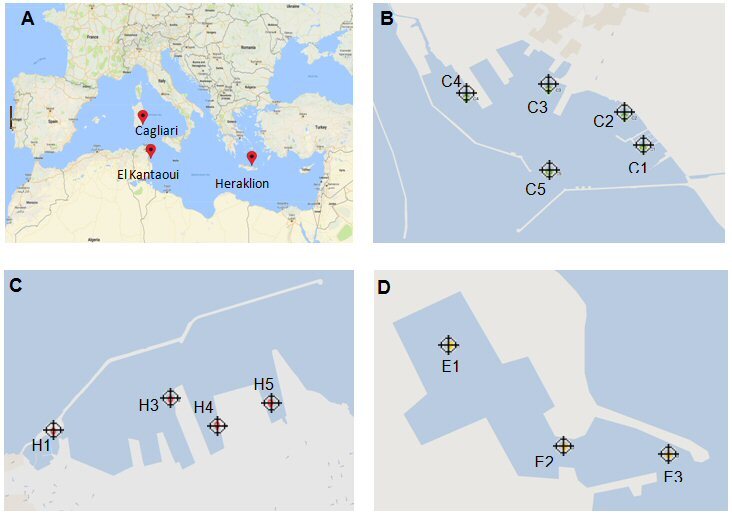
The three Mediterranean touristic ports (A) and location of the sampling stations in Cagliari (B), Heraklion (C) and El Kantaoui (D)

Three Mediterranean touristic ports were selected as study sites and they are presented in Fig. 1. The port of Cagliari (Fig. 1B) is a large port (2.07 km^2^) located on the southern coast of Sardinia (Italy). The port of Heraklion (Fig. 1C) is medium-sized (0.87 km^2^) and it is located on the northern coast of Crete (Greece). The port of El Kantaoui (Fig. 1D) is a small touristic marina (0.04 km^2^) on the eastern Tunisian coast. Both the ports of Heraklion and Cagliari host a leisure marina, large passenger, cruise and cargo vessels, while El Kantaoui port offers moorings only for smaller fishing boats, luxury yachts and boats for sporting activities. Heraklion port has also a shipyard section.

Sampling stations were selected in order to achieve good spatial coverage in each port and also to represent sectors with different uses according to the Water Management Units in MAPMED Action Plans (MAPMED 2015). Four stations were selected in the port of Heraklion, five stations in the port of Cagliari and three stations in the port of El Kantaoui (Table 1).

### Coordinates

33.724 and 41.311 Latitude; 7.954 and 27.598 Longitude.

## Taxonomic coverage

### Description

The dataset includes information on macrobenthic assemblages found in the three Mediterranean touristic ports belonging to the following 12 phyla: Annelida, Mollusca, Arthropoda, Echinodermata, Sipuncula, Nemertea, Cnidaria, Phoronida, Chordata, Foraminifera, Platyhelminthes and Priapulida (Table 2).

**Figure 2.**
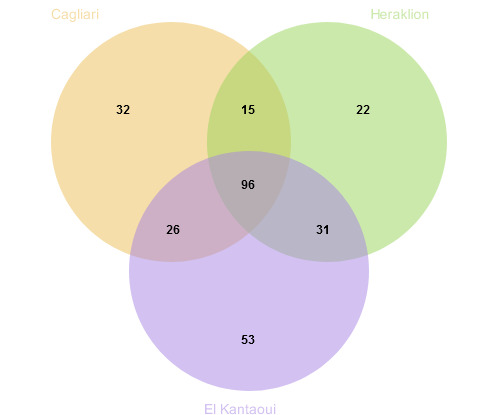
Chart showing number of common and unique taxa in the three Mediterranean ports (Cagliari, Heraklion and El Kantaoui).

A total of 46,187 individuals were identified down to the lowest possible taxonomic level, from which 15,535 were found in Cagliari port, 11,571 in Heraklion port and 19,081 in El Kantaoui port. A total of 277 macrofaunal taxa were identified in the three ports, from which 32 were present exclusively in Cagliari port, 22 were found only in Heraklion port and 53 were found only in El Kantaoui port. A total of 96 taxa were common between all three Mediterranean ports (Fig. 2). The highest number of taxa in total was found in El Kantaoui port (206), while Cagliari and Heraklion ports had 170 and 165 taxa in total, respectively.

**Figure 3.**
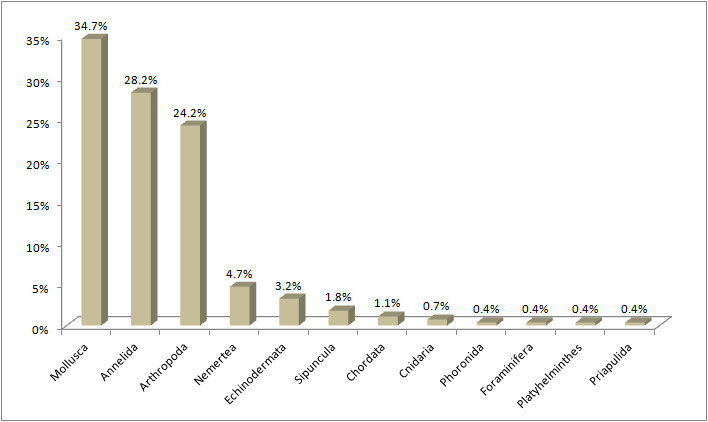
Distribution of the different phyla found in the three Mediterranean ports (Cagliari, Heraklion and El Kantaoui).

Mollusca were the most abundant group (34.7%), Annelida (28.2%) and Arthropoda (24.2%) were also highly represented, while each of the remaining groups contributed less than 5% in the benthic assemblages studied (Fig. 3).

**Figure 4.**
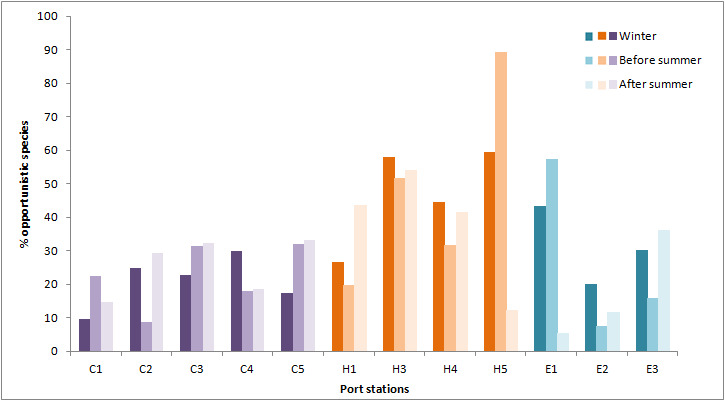
Percentage (%) of opportunistic taxa abundances in the different stations of Cagliari port (purple: C1, C2, C3, C4 and C5), Heraklion port (orange: H1, H3, H4 and H5) and El Kantaoui (blue: E1, E2 and E3) during the three differerent sampling seasons (dark colours: winter, medium colours: before summer, light colours: after summer).

The percentage of opportunistic taxa abundances (i.e. short-lived taxa often characterising disturbed or stressed habitats) was calculated for each station as the percentage of abundances for Capitellidae, Cirratulidae, Spionidae and Oligochaeta taxa (Pearson and Rosenberg 1978, Munari et al. 2005) in the total sample (Fig. 4). The highest percentages (> 50%) of opportunistic taxa abundances were found in Heraklion port at stations H3 (passenger ships) during all seasons and at H5 (shipyard station) in winter and especially before summer. Additionally, in El Kantaoui station E1 (leisure boats), the percentage of opportunistic taxa abundance increased before summer. The percentage of opportunists in Cagliari was generally lower than in the other ports.

Additional statistical analysis has been applied to the species composition matrices of the specific dataset by Chatzinikolaou et al. (2018) and Dimitriou et al. (2020) in order to explore the multivariate patterns of benthic assemblages and to calculate benthic diversity and biotic indices for the assessment of the ecological status of the habitats. A detailed comparison of macrobenthic biodiversity amongst the different locations - sectors - seasons indicated significant differences between ports and between sectors in each port, while seasonal differences were not apparent (Chatzinikolaou et al. 2018).

## Temporal coverage

**Data range:** 2012-2-13 – 2012-9-25.

### Notes

Three seasonal sampling campaigns were carried out during 2012: one in winter (February), one in spring before the beginning of the touristic season (May) and one in late summer after the touristic season (September).

## Collection data

### Collection name

Benthic communities and environmental parameters in three Mediterranean ports (Sardinia, Crete and Tunisia).

### Specimen preservation method

5% formaldehyde buffered with seawater.

### Curatorial unit

Hellenic Centre for Marine Research (HCMR), Institute of Marine Biology, Biotechnology and Aquaculture (IMBBC), Heraklion, Crete, Greece.

## Usage licence

### Usage licence

Open Data Commons Attribution License

### IP rights notes

This work is licensed under a Creative Commons Attribution (CC-BY) 4.0 License. All data in the database can be freely used provided it is fully cited even if only partially used.

## Data resources

### Data package title

Benthic communities and environmental parameters in three Mediterranean ports (Sardinia, Crete and Tunisia).

### Resource link

https://www.gbif.org/dataset/9b11f305-fb7a-4a65-826e-7fb97af06e5f

### Alternative identifiers

http://ipt.medobis.eu/resource?r=mapmed_ports

### Number of data sets

1

### Data set 1.

#### Data set name

Benthic communities and environmental parameters in three Mediterranean ports (Sardinia, Crete and Tunisia).

#### Data format

Darwin Core Archive.

#### Character set

UTF-8

#### Download URL

http://ipt.medobis.eu/resource?r=mapmed_ports

#### Description

The dataset is available via the MedOBIS (Mediterranean node of Ocean Biodiversity Information System), Integrated Publishing Toolkit (IPT) which has been established through the LifeWatchGreece Research Infrastructure and is hosted in the Institute of Marine Biology, Biotechnology and Aquaculture (IMBBC) of the Hellenic Centre for Marine Research (HCMR). The data are also harvested by and made available through the Ocean Biodiversity Information System (OBIS). The dataset is available as a DarwinCoreArchive and all fields are mapped to DarwinCore terms.

This publication refers to the most recent version of the dataset available through the IPT server or MedOBIS. Future changes to the dataset due to quality control activities might change its content or structure.

The current publication refers to the "occurrence" source file (txt file) that is associated with the particular dataset. Additional details about the sampling events can be found in the "event" source file (txt file) associated with the same dataset.

**Data set 1.** 

Column label	Column description
id	A unique identifier for the record within the dataset or collection, auto-incrementing number automatically added by the system (same with eventID).
eventID	An identifier for the set of information associated with an Event (something that occurs at a place and time).
samplingProtocol	The description of the method or protocol used for sample collection.
eventDate	The date-time or interval during which an Event occurred.
year	The four-digit year in which the Event occurred, according to the Common Era Calendar.
month	The integer month in which the Event occurred.
day	The integer day of the month on which the Event occurred.
eventRemarks	Comments or notes about the Event.
locationID	An identifier for the set of location information (station name).
locality	The specific description of the place.
minimumDepthInMetres	The lesser depth of a range of depth below the local surface, in metres.
maximumDepthInMetres	The greater depth of a range of depth below the local surface, in metres.
decimalLatitude	The geographic latitude (in decimal degrees, using the spatial reference system given in geodeticDatum) of the geographic centre of a Location. Positive values are north of the Equator, negative values are south of it. Legal values lie between -90 and 90, inclusive.
decimalLongitude	The geographic longitude (in decimal degrees, using the spatial reference system given in geodeticDatum) of the geographic centre of a Location. Positive values are east of the Greenwich Meridian, negative values are west of it. Legal values lie between -180 and 180, inclusive.
geodeticDatum	The ellipsoid, geodetic datum or spatial reference system (SRS) upon which the geographic coordinates given in decimalLatitude and decimalLongitude are based.
coordinateUncertaintyInMetres	The horizontal distance (in metres) from the given decimalLatitude and decimalLongitude describing the smallest circle containing the whole of the Location.
georeferenceProtocol	A description or reference to the methods used to determine the spatial footprint, coordinates and uncertainties.
institutionCode	The name (or acronym) in use by the institution having custody of the object (s) or information referred to in the record.
collectionCode	An identifier for the collection or dataset from which the record was derived.
basisOfRecord	The specific nature of the data record.
occurrenceID	An identifier for the Occurrence (as opposed to a particular digital record of the occurrence).
catalogNumber	An identifier (preferably unique) for the record within the dataset or collection.
individualCount	The number of individuals represented present at the time of the Occurrence.
occurrenceStatus	A statement about the presence or absence of a Taxon at a Location.
scientificNameID	An identifier for the nomenclatural (not taxonomic) details of a scientific name (Isid of WORMS).
scientificName	The full scientific name, not including authorship.
kingdom	The full scientific name of the kingdom in which the taxon is classified.
phylum	The full scientific name of the phylum in which the taxon is classified.
class	The full scientific name of the class in which the taxon is classified.
order	The full scientific name of the order in which the taxon is classified.
family	The full scientific name of the family in which the taxon is classified.
genus	The full scientific name of the genus in which the taxon is classified.
specificEpithet	The species epithet of the scientificName.
scientificNameAuthorship	The authorship information for the scientificName formatted according to the conventions of the applicable nomenclaturalCode.
nomenclaturalCode	The nomenclatural code (or codes in the case of an ambiregnal name) under which the scientificName is constructed.
taxonRemarks	Comments or notes about the taxon or name in the original dataset file.

## Figures and Tables

**Table 1.** Maximum depth (m), use sectors and coordinates of all sampling stations in the Meditarranean ports of Cagliari, Heraklion and El Kantaoui.

**Port**	**Station**	**Depth (m)**	**Port sector**	**Coordinates**
Cagliari	C1	7.8	Leisure/fishing	39°12'12.2''N; 09°07'25.1''E
	C2	4.5	Leisure/fishing	39°12'23.0''N; 09°07'16.7''E
	C3	8.3	Leisure/fishing	39°12'34.0''N; 09°06'45.9''E
	C4	13.5	Passenger/cargo ships	39°12'29.7''N; 09°06'14.8''E
	C5	11.4	Intense maritime traffic	39°12'01.7''N; 09°06'45.2''E
Heraklion	H1	3.7	Leisure/fishing	35°20'38.7''N; 25°08'12.4''E
	H3	19.5	Passenger ships	35°20'45.6''N; 25°08'43.3''E
	H4	10.5	Cargo ships	35°20'39.5''N; 25°08'55.5''E
	H5	19.0	Shipyard	35°20'42.9''N; 25°09'08.6''E
El Kantaoui	E1	2.5	Leisure/fishing	35°53'39.9''N; 10°35'52.1''E
	E2	4.0	Leisure/fishing	35°53'34.6''N; 10°35'58.9''E
	E3	3.2	Leisure/fishing	35°53'34.1''N; 10°36'05.2''E

**Table 2.** List of taxa (phylum, class, family and species name) found in the three Mediterranean touristic ports.

**Phylum**	**Class**	**Family**	**Species**	**Cagliari**	**Heraklion**	**El Kantaoui**
Annelida	Polychaeta	Ampharetidae	*Amageadspersa*	x		
Annelida	Polychaeta	Amphinomidae	*Chloeiaviridis*		x	
Annelida	Polychaeta	Amphinomidae	*Eurythoecomplanata*	x		
Annelida	Polychaeta	Capitellidae	*Capitellacapitata*	x	x	x
Annelida	Polychaeta	Capitellidae	*Capitellagiardi*		x	x
Annelida	Polychaeta	Capitellidae	*Heteromastusfiliformis*	x	x	x
Annelida	Polychaeta	Capitellidae	*Notomastuslatericeus*	x	x	x
Annelida	Polychaeta	Chaetopteridae	*Spiochaetopteruscostarum*	x		x
Annelida	Polychaeta	Cirratulidae	*Aphelochaetafiliformis*	x	x	x
Annelida	Polychaeta	Cirratulidae	*Aphelochaetamarioni*	x	x	x
Annelida	Polychaeta	Cirratulidae	*Cirriformiatentaculata*	x	x	x
Annelida	Polychaeta	Cirratulidae	*Tharyxkillariensis*	x	x	x
Annelida	Polychaeta	Cossuridae	*Cossurasoyeri*	x	x	
Annelida	Polychaeta	Dorvilleidae	*Dorvilleaatlantica*			x
Annelida	Polychaeta	Dorvilleidae	*Dorvillearubrovittata*	x	x	
Annelida	Polychaeta	Dorvilleidae	*Protodorvilleakefersteini*		x	x
Annelida	Polychaeta	Dorvilleidae	*Schistomeringosrudolphi*	x	x	x
Annelida	Polychaeta	Eunicidae	*Eunicevittata*	x	x	x
Annelida	Polychaeta	Eunicidae	*Lysidiceunicornis*		x	
Annelida	Polychaeta	Flabelligeridae	*Piromiseruca*		x	x
Annelida	Polychaeta	Glyceridae	*Glyceralapidum*	x	x	x
Annelida	Polychaeta	Glyceridae	*Glyceratesselata*	x		
Annelida	Polychaeta	Glyceridae	*Glyceratridactyla*	x	x	x
Annelida	Polychaeta	Goniadidae	*Goniadaemerita*		x	
Annelida	Polychaeta	Hesionidae	*Psamathefusca*	x	x	x
Annelida	Polychaeta	Lumbrineridae	*Hilbignerisgracilis*	x	x	x
Annelida	Polychaeta	Lumbrineridae	*Lumbrinerislatreilli*	x	x	x
Annelida	Polychaeta	Lumbrineridae	*Scoletomafunchalensis*		x	
Annelida	Polychaeta	Magelonidae	*Magelonaminuta*			x
Annelida	Polychaeta	Maldanidae	*Euclymenepalermitana*	x		x
Annelida	Polychaeta	Maldanidae	*Praxillellaaffinis*	x	x	
Annelida	Polychaeta	Maldanidae	*Praxillellagracilis*	x	x	
Annelida	Polychaeta	Melinnidae	*Melinnapalmata*	x	x	x
Annelida	Polychaeta	Nephtyidae	*Nephtyscaeca*		x	x
Annelida	Polychaeta	Nephtyidae	*Nephtyshystricis*	x	x	x
Annelida	Polychaeta	Nephtyidae	*Nephtysincisa*	x		
Annelida	Polychaeta	Nereididae	*Neanthesacuminata*	x	x	x
Annelida	Polychaeta	Nereididae	*Nereisrava*			x
Annelida	Polychaeta	Nereididae	*Nereiszonata*		x	
Annelida	Polychaeta	Onuphidae	*Aponuphisbilineata*	x		x
Annelida	Polychaeta	Onuphidae	*Aponuphisbrementi*		x	x
Annelida	Polychaeta	Onuphidae	*Diopatraneapolitana*	x	x	x
Annelida	Polychaeta	Opheliidae	*Polyophthalmuspictus*	x	x	x
Annelida	Polychaeta	Orbiniidae	*Phylofoetida*	x	x	x
Annelida	Polychaeta	Orbiniidae	Scoloplos (Scoloplos) armiger	x	x	x
Annelida	Polychaeta	Oweniidae	*Oweniafusiformis*	x	x	x
Annelida	Polychaeta	Paraonidae	Aricidea (Aricidea) fragilis	x	x	x
Annelida	Polychaeta	Paraonidae	*Levinseniagracilis*	x	x	
Annelida	Polychaeta	Paraonidae	*Paradoneislyra*	x	x	x
Annelida	Polychaeta	Pectinariidae	*Lagiskoreni*	x	x	x
Annelida	Polychaeta	Phyllodocidae	*Eteonelonga*			x
Annelida	Polychaeta	Phyllodocidae	*Mystapicta*		x	
Annelida	Polychaeta	Phyllodocidae	*Phyllodoce* sp.	x		
Annelida	Polychaeta	Pilargidae	*Ancistrosyllisgroenlandica*	x		
Annelida	Polychaeta	Poecilochaetidae	*Poecilochaetusserpens*		x	x
Annelida	Polychaeta	Polynoidae	*Harmothoeimpar*	x	x	
Annelida	Polychaeta	Polynoidae	*Harmothoespinifera*		x	x
Annelida	Polychaeta	Sabellidae	*Amphiglenamediterranea*	x		
Annelida	Polychaeta	Sabellidae	*Dialychonedunerificta*			x
Annelida	Polychaeta	Sabellidae	*Pseudopotamillareniformis*	x	x	x
Annelida	Polychaeta	Scalibregmatidae	*Polyphysiacrassa*			x
Annelida	Polychaeta	Serpulidae	*Serpulavermicularis*	x	x	x
Annelida	Polychaeta	Spionidae	*Polydoraciliata*			x
Annelida	Polychaeta	Spionidae	*Prionospiocirrifera*	x		x
Annelida	Polychaeta	Spionidae	*Prionospiomalmgreni*	x	x	x
Annelida	Polychaeta	Spionidae	*Spiofilicornis*	x	x	x
Annelida	Polychaeta	Sternaspidae	*Sternaspisscutata*	x	x	x
Annelida	Polychaeta	Syllidae	*Exogone* sp.	x		
Annelida	Polychaeta	Syllidae	*Sphaerosyllishystrix*	x	x	x
Annelida	Polychaeta	Syllidae	*Syllisarmillaris*	x	x	x
Annelida	Polychaeta	Syllidae	*Syllisgarciai*			x
Annelida	Polychaeta	Syllidae	*Syllishyalina*	x	x	x
Annelida	Polychaeta	Syllidae	*Syllisprolifera*	x	x	x
Annelida	Polychaeta	Terebellidae	*Laniceconchilega*	x		x
Annelida	Polychaeta	Terebellidae	*Pistacristata*	x	x	x
Annelida	Polychaeta	Trichobranchidae	*Terebellidesstroemii*	x		
Annelida	Polychaeta		Echiura ind.	x	x	x
Annelida	Clitellata		Oligochaeta ind.	x	x	x
Arthropoda	Arachnida	Pontarachnidae	*Pontarachnapunctulum*	x		
Arthropoda	Insecta		Insecta ind.	x	x	x
Arthropoda	Malacostraca	Ampeliscidae	*Ampeliscadiadema*	x		
Arthropoda	Malacostraca	Ampeliscidae	*Ampeliscaledoyeri*	x		
Arthropoda	Malacostraca	Amphilochidae	*Apolochusneapolitanus*			x
Arthropoda	Malacostraca	Anthuridae	Anthuridae ind.	x	x	x
Arthropoda	Malacostraca	Anthuridae	*Cyathuracarinata*			x
Arthropoda	Malacostraca	Aoridae	*Aora* sp.	x		
Arthropoda	Malacostraca	Aoridae	*Aoragracilis*	x	x	
Arthropoda	Malacostraca	Aoridae	*Aoraspinicornis*		x	x
Arthropoda	Malacostraca	Aoridae	Aoridae ind.	x	x	x
Arthropoda	Malacostraca	Aoridae	*Autonoespiniventris*	x		x
Arthropoda	Malacostraca	Aoridae	*Lemboswebsteri*	x	x	
Arthropoda	Malacostraca	Aoridae	*Microdeutopus* sp.	x		x
Arthropoda	Malacostraca	Aoridae	*Microdeutopusanomalus*		x	x
Arthropoda	Malacostraca	Aoridae	*Microdeutopusstationis*		x	
Arthropoda	Malacostraca	Aoridae	*Microdeutopusversiculatus*	x		
Arthropoda	Malacostraca	Apseudidae	*Apseudopsismediterraneus*	x	x	x
Arthropoda	Malacostraca	Atylidae	*Nototropisguttatus*		x	
Arthropoda	Malacostraca	Bodotriidae	*Bodotriascorpioides*	x		x
Arthropoda	Malacostraca	Bodotriidae	*Iphinoeserrata*	x	x	x
Arthropoda	Malacostraca	Bodotriidae	*Iphinoetenella*	x		x
Arthropoda	Malacostraca	Bodotriidae	*Iphinoetrispinosa*	x	x	x
Arthropoda	Malacostraca	Bodotriidae	*Vaunthompsoniacristata*		x	x
Arthropoda	Malacostraca	Calliopiidae	*Apherusamediterranea*		x	
Arthropoda	Malacostraca	Caprellidae	*Liropuselongatus*		x	
Arthropoda	Malacostraca	Caprellidae	*Phtisicamarina*	x	x	x
Arthropoda	Malacostraca	Caprellidae	*Pseudoliriuskroyeri*	x	x	x
Arthropoda	Malacostraca	Corophiidae	*Apocorophiumacutum*	x	x	x
Arthropoda	Malacostraca	Corophiidae	*Leptocheirus* sp.		x	
Arthropoda	Malacostraca	Corophiidae	*Medicorophiumminimum*	x		
Arthropoda	Malacostraca	Corophiidae	*Medicorophiumruncicorne*	x	x	x
Arthropoda	Malacostraca	Dexaminidae	*Dexaminespinosa*		x	x
Arthropoda	Malacostraca	Diastylidae	*Diastylisrugosa*		x	
Arthropoda	Malacostraca	Gammaridae	Gammaridae ind.	x	x	x
Arthropoda	Malacostraca	Gnathiidae	*Gnathia* sp.	x	x	x
Arthropoda	Malacostraca	Holognathidae	*Cleantisprismatica*		x	x
Arthropoda	Malacostraca	Hyalidae	*Apohyaleperieri*	x		
Arthropoda	Malacostraca	Hyalidae	*Parhyaleaquilina*	x		
Arthropoda	Malacostraca	Idoteidae	*Idotea* sp.		x	x
Arthropoda	Malacostraca	Ischyroceridae	*Ericthoniuspunctatus*		x	x
Arthropoda	Malacostraca	Ischyroceridae	*Jassamarmorata*	x	x	
Arthropoda	Malacostraca	Leptocheliidae	*Chondrocheliasavignyi*	x	x	x
Arthropoda	Malacostraca	Leucothoidae	*Leucothoeincisa*	x	x	x
Arthropoda	Malacostraca	Maeridae	*Elasmopusrapax*	x	x	x
Arthropoda	Malacostraca	Maeridae	*Maeragrossimana*	x	x	x
Arthropoda	Malacostraca	Melitidae	*Melita hergensis*	x	x	x
Arthropoda	Malacostraca	Microprotopidae	*Microprotopusmaculatus*			x
Arthropoda	Malacostraca	Nannastacidae	Cumella (Cumella) pygmaea	x	x	x
Arthropoda	Malacostraca	Nannastacidae	*Nannastacus* sp.			x
Arthropoda	Malacostraca	Nuuanuidae	*Gammarellafucicola*		x	x
Arthropoda	Malacostraca	Oedicerotidae	*Kroyeracarinata*	x		
Arthropoda	Malacostraca	Oedicerotidae	*Perioculodeslongimanus*	x	x	x
Arthropoda	Malacostraca	Oedicerotidae	*Pontocratesarenarius*		x	
Arthropoda	Malacostraca	Oedicerotidae	*Synchelidiumhaplocheles*	x		x
Arthropoda	Malacostraca	Oedicerotidae	*Synchelidiummaculatum*	x	x	
Arthropoda	Malacostraca	Paranthuridae	*Paranthurajaponica*			x
Arthropoda	Malacostraca	Photidae	*Cerapopsislongipes*	x		
Arthropoda	Malacostraca	Sphaeromatidae	*Dynamenebidentata*		x	x
Arthropoda	Malacostraca	Sphaeromatidae	*Sphaeroma* sp.	x		
Arthropoda	Malacostraca	Stenothoidae	*Stenothoeelachista*			x
Arthropoda	Malacostraca	Tanaididae	*Tanaisdulongii*	x	x	x
Arthropoda	Malacostraca		Amphipoda ind.	x	x	x
Arthropoda	Malacostraca		Isopoda ind.	x	x	x
Arthropoda	Malacostraca		Tanaidacea ind.		x	
Arthropoda	Pycnogonida		Pycnogonida ind.1		x	
Arthropoda	Pycnogonida		Pycnogonida ind.2		x	
Chordata	Ascidiacea		Ascidiacea ind.			x
Chordata	Ascidiacea	Styelidae	*Botryllus* sp.			x
Chordata			Pisces ind.	x		
Cnidaria	Anthozoa		Anthozoa ind.	x	x	x
Cnidaria	Hydrozoa		Hydrozoa ind.		x	x
Echinodermata	Asteroidea		Asteroidea ind.	x		x
Echinodermata	Holothuroidea	Cucumariidae	*Trachythyone* sp.	x		
Echinodermata	Holothuroidea		Holothuroidea ind.1	x	x	
Echinodermata	Holothuroidea		Holothuroidea ind.2			x
Echinodermata	Ophiuroidea	Amphiuridae	*Amphiurachiajei*	x	x	x
Echinodermata	Ophiuroidea	Ophiodermatidae	*Ophiodermalongicaudum*	x	x	x
Echinodermata	Ophiuroidea		Ophiuroidea ind.1	x	x	x
Echinodermata	Ophiuroidea		Ophiuroidea ind.2	x	x	x
Echinodermata	Ophiuroidea		Ophiuroidea ind.3	x		
Foraminifera			Foraminifera ind.	x		x
Mollusca	Bivalvia	Arcidae	*Arcatetragona*	x		x
Mollusca	Bivalvia	Cardiidae	*Cerastodermaglaucum*		x	x
Mollusca	Bivalvia	Cardiidae	*Papillicardiumminimum*	x	x	x
Mollusca	Bivalvia	Cardiidae	*Papillicardiumpapillosum*	x		x
Mollusca	Bivalvia	Cardiidae	*Parvicardiumexiguum*	x	x	x
Mollusca	Bivalvia	Corbulidae	*Varicorbulagibba*	x	x	
Mollusca	Bivalvia	Lasaeidae	*Kurtiellabidentata*	x	x	x
Mollusca	Bivalvia	Lasaeidae	*Litigiellaglabra*	x	x	x
Mollusca	Bivalvia	Lucinidae	*Ctenadecussata*	x	x	x
Mollusca	Bivalvia	Lucinidae	*Loripesorbiculatus*	x	x	x
Mollusca	Bivalvia	Lucinidae	*Loripinusfragilis*		x	
Mollusca	Bivalvia	Lucinidae	*Lucinelladivaricata*	x	x	x
Mollusca	Bivalvia	Lucinidae	*Myrteaspinifera*	x	x	x
Mollusca	Bivalvia	Mytilidae	*Modiolusadriaticus*	x	x	x
Mollusca	Bivalvia	Mytilidae	*Modiolusbarbatus*	x		x
Mollusca	Bivalvia	Mytilidae	*Musculussubpictus*	x	x	x
Mollusca	Bivalvia	Mytilidae	*Mytilusedulis*	x		
Mollusca	Bivalvia	Nuculanidae	*Lembuluspella*	x	x	x
Mollusca	Bivalvia	Nuculidae	*Nuculanitidosa*	x	x	
Mollusca	Bivalvia	Pharidae	*Pharuslegumen*			x
Mollusca	Bivalvia	Semelidae	*Abraalba*	x	x	x
Mollusca	Bivalvia	Semelidae	*Abraprismatica*	x		
Mollusca	Bivalvia	Solemyidae	*Solemyatogata*			x
Mollusca	Bivalvia	Tellinidae	*Asbjornseniapygmaea*			x
Mollusca	Bivalvia	Tellinidae	*Gastranafragilis*			x
Mollusca	Bivalvia	Tellinidae	*Macomopsiscumana*			x
Mollusca	Bivalvia	Tellinidae	*Moerelladonacina*	x	x	x
Mollusca	Bivalvia	Tellinidae	*Peronidiaalbicans*	x	x	x
Mollusca	Bivalvia	Thraciidae	*Thraciaphaseolina*	x		
Mollusca	Bivalvia	Veneridae	*Chameleagallina*		x	x
Mollusca	Bivalvia	Veneridae	*Dosinialupinus*	x	x	x
Mollusca	Bivalvia	Veneridae	*Gouldiaminima*	x	x	x
Mollusca	Bivalvia	Veneridae	*Pitarrudis*	x		
Mollusca	Bivalvia	Veneridae	*Ruditapesdecussatus*	x		x
Mollusca	Bivalvia	Veneridae	*Venerupislucens*	x	x	x
Mollusca	Gastropoda	Bullidae	*Bullastriata*	x		x
Mollusca	Gastropoda	Caecidae	*Caecumsubannulatum*		x	x
Mollusca	Gastropoda	Cerithiidae	*Bittiumlatreillii*	x	x	x
Mollusca	Gastropoda	Cerithiidae	*Bittiumreticulatum*		x	x
Mollusca	Gastropoda	Cerithiidae	*Cerithiumprotractum*			x
Mollusca	Gastropoda	Cerithiidae	*Cerithiumscabridum*			x
Mollusca	Gastropoda	Cerithiidae	*Cerithiumvulgatum*			x
Mollusca	Gastropoda	Cerithiopsidae	*Cerithiopsisminima*			x
Mollusca	Gastropoda	Conidae	*Conusventricosus*	x		
Mollusca	Gastropoda	Epitoniidae	*Epitoniumclathrus*			x
Mollusca	Gastropoda	Eulimidae	*Eulimabilineata*	x		
Mollusca	Gastropoda	Eulimidae	*Parviorisibizenca*			x
Mollusca	Gastropoda	Granulinidae	*Granulinamarginata*	x		x
Mollusca	Gastropoda	Mangeliidae	*Mangeliapaciniana*			x
Mollusca	Gastropoda	Mangeliidae	*Sorgenfreispirabrachystoma*		x	x
Mollusca	Gastropoda	Muricidae	*Hexaplextrunculus*	x	x	x
Mollusca	Gastropoda	Muricidae	*Nucellalapillus*			x
Mollusca	Gastropoda	Nassariidae	*Tritiacorniculum*	x		x
Mollusca	Gastropoda	Nassariidae	*Tritiareticulata*	x	x	x
Mollusca	Gastropoda	Nassariidae	*Tritiavaricosa*	x	x	x
Mollusca	Gastropoda	Naticidae	*Neveritajosephinia*		x	x
Mollusca	Gastropoda	Neritidae	*Smaragdiaviridis*			x
Mollusca	Gastropoda	Omalogyridae	*Ammonicerarota*	x		x
Mollusca	Gastropoda	Phasianellidae	*Tricoliapullus*			x
Mollusca	Gastropoda	Phasianellidae	*Tricoliaspeciosa*	x		x
Mollusca	Gastropoda	Phasianellidae	*Tricoliatenuis*	x		x
Mollusca	Gastropoda	Pyramidellidae	*Chrysallidaindistincta*		x	x
Mollusca	Gastropoda	Pyramidellidae	*Eulimellaacicula*		x	
Mollusca	Gastropoda	Pyramidellidae	*Megastomiaconoidea*	x	x	x
Mollusca	Gastropoda	Pyramidellidae	*Odostomiaunidentata*		x	
Mollusca	Gastropoda	Pyramidellidae	*Ondinadiaphana*	x	x	x
Mollusca	Gastropoda	Pyramidellidae	*Partheninaterebellum*		x	x
Mollusca	Gastropoda	Pyramidellidae	*Pyrgiscusrufus*			x
Mollusca	Gastropoda	Pyramidellidae	*Turbonillagradata*		x	x
Mollusca	Gastropoda	Pyramidellidae	*Turbonillamicans*			x
Mollusca	Gastropoda	Pyramidellidae	*Turbonillamultilirata*			x
Mollusca	Gastropoda	Pyramidellidae	*Turbonillapumila*			x
Mollusca	Gastropoda	Raphitomidae	*Raphitomaphilberti*		x	x
Mollusca	Gastropoda	Retusidae	*Retusatruncatula*		x	x
Mollusca	Gastropoda	Retusidae	*Retusaumbilicata*			x
Mollusca	Gastropoda	Rissoidae	*Alvaniaalgeriana*			x
Mollusca	Gastropoda	Rissoidae	*Alvaniacimex*	x	x	x
Mollusca	Gastropoda	Rissoidae	*Alvaniadiscors*			x
Mollusca	Gastropoda	Rissoidae	*Alvaniamamillata*	x	x	x
Mollusca	Gastropoda	Rissoidae	*Manzoniacrassa*		x	
Mollusca	Gastropoda	Rissoidae	*Pusillinamarginata*	x		x
Mollusca	Gastropoda	Rissoidae	*Pusillinaradiata*	x	x	x
Mollusca	Gastropoda	Rissoidae	*Rissoaaartseni*			x
Mollusca	Gastropoda	Rissoidae	*Rissoasplendida*		x	x
Mollusca	Gastropoda	Skeneidae	*Skeneaserpuloides*			x
Mollusca	Gastropoda	Trochidae	*Gibbulaardens*	x		x
Mollusca	Gastropoda	Trochidae	*Gibbulamagus*	x		
Mollusca	Gastropoda	Trochidae	*Jujubinusgravinae*	x		
Mollusca	Gastropoda	Trochidae	*Jujubinuskarpathoensis*		x	
Mollusca	Gastropoda	Trochidae	*Jujubinusmontagui*	x		
Mollusca	Gastropoda	Trochidae	*Jujubinusstriatus*	x		x
Mollusca	Gastropoda	Trochidae	*Jujubinusunidentatus*			x
Mollusca	Gastropoda	Trochidae	*Steromphalaadansonii*			x
Mollusca	Gastropoda	Trochidae	*Steromphalavaria*		x	x
Mollusca	Scaphopoda	Dentaliidae	*Antalisdentalis*	x	x	
Mollusca	Scaphopoda	Fustiariidae	*Fustiariarubescens*			x
Nemertea	Hoplonemertea	Amphiporidae	*Amphiporus* sp.		x	x
Nemertea	Hoplonemertea	Carcinonemertidae	*Carcinonemertescarcinophila*			x
Nemertea	Hoplonemertea	Drepanophoridae	*Drepanophorus* sp.			x
Nemertea	Hoplonemertea	Oerstediidae	*Oerstediadorsalis*			x
Nemertea	Hoplonemertea	Ototyphlonemertidae	*Ototyphlonemertesduplex*			x
Nemertea	Hoplonemertea	Prosorhochmidae	*Prosorhochmus* sp.	x	x	
Nemertea	Hoplonemertea	Tetrastemmatidae	*Tetrastemma* sp.			x
Nemertea	Palaeonemertea	Cephalotrichidae	*Cephalothrix* sp.		x	
Nemertea	Pilidiophora	Lineidae	*Micrura* sp.	x	x	
Nemertea			Nemertea ind.1	x	x	
Nemertea			Nemertea ind.2	x	x	x
Nemertea			Nemertea ind.3			x
Nemertea			Nemertea ind.4	x	x	x
Phoronida			Phoronida ind.	x		x
Platyhelminthes	Turbellaria		Turbellaria ind.			x
Priapulida			Priapulida ind.	x		
Sipuncula	Phascolosomatidea	Phascolosomatidae	*Phascolosoma* sp.	x	x	x
Sipuncula	Sipunculidea	Sipunculidae	Sipunculus (Sipunculus) nudus			x
Sipuncula			Sipuncula ind.1	x	x	x
Sipuncula			Sipuncula ind.2			x
Sipuncula			Sipuncula ind.3	x	x	x

## References

[B6842230] Chatzinikolaou E., Arvanitidis C. (2017). Benthic communities and environmental parameters in three Mediterranean ports (Sardinia, Crete, Tunisia).

[B6829880] Chatzinikolaou E., Mandalakis M., Damianidis P., Dailianis T., Gambineri S., Rossano C., Scapini F., Carucci A., Arvanitidis C. (2018). Spatio-temporal benthic biodiversity patterns and pollution pressure in three Mediterranean touristic ports. Science of the Total Environment.

[B6814855] Darbra R. M., Ronza A., Stojanovic T. A., Wooldridge C., Casal J. (2005). A procedure for identifying significant environmental aspects in sea ports. Marine Pollution Bulletin.

[B6829894] Dimitriou P., Chatzinikolaou E., Arvanitidis C. (2020). Ecological status assessment based on benthic macrofauna of three Mediterranean ports: Comparisons across seasons, activities and regions. Marine Pollution Bulletin.

[B6814865] Eurostat Maritime transport statistics - short sea shipping of goods. http://ec.europa.eu/eurostat/statistics-explained/index.php/Maritime_transport_statistics_-_short_sea_shipping_of_goods.

[B6814873] Gray J. S., Elliot M. (2010). Ecology of marine sediments: From science to management.

[B6814881] Mandal S., Harkantra S. N. (2013). Changes in the soft-bottom macrobenthic diversity and community structure from the ports of Mumbai, India. Environmental Monitoring Assessment.

[B6814899] Munari C., Rossi R., Mistri M. (2005). Temporal trends in macrobenthos community structure and redundancy in a shallow coastal lagoon (Valli di Comacchio, Northern Adriatic Sea). Hydrobiologia.

[B6814890] Pearson T. H., Rosenberg R. (1978). Macrobenthic succession in relation to organic enrichment and pollution of the marine environment. Oceanography and Marine Biology - An Annual Review.

[B6814846] REMPEC (2008). Regional Marine Pollution Emergency Response Centre for the Mediterranean Sea. Study of maritime traffic flows in the Mediterranean Sea. Final Report standards in Southern California marine waters. Water Research.

